# Association between educational level and smoking cessation in an 11-year follow-up study of a national health survey

**DOI:** 10.1177/1403494821993721

**Published:** 2021-03-01

**Authors:** Otto Ruokolainen, Tommi Härkänen, Jouni Lahti, Ari Haukkala, Markku Heliövaara, Ossi Rahkonen

**Affiliations:** 1 Finnish Institute for Health and Welfare, Finland; 2 Faculty of Social Sciences, University of Helsinki, Finland; 3 Helsinki Collegium for Advanced Studies, University of Helsinki, Finland; 4 Department of Public Health, University of Helsinki, Finland

**Keywords:** Smoking, tobacco use, smoking cessation, education, socio-economic position, longitudinal studies, health inequalities, population based

## Abstract

**Aims:** There is a lack of longitudinal, population-based studies on the association between education and smoking cessation. A more thorough examination of this association is needed to address inequalities in smoking. **Methods:** The longitudinal Health 2000 Survey and Health 2011 Survey, representing the Finnish population aged ⩾30 years, were analysed. Of the 1352 baseline daily smokers, 945 (70%) provided a smoking status at the follow-up. The analytic sample size was 884 (excluding the follow-up occasional smokers). Self-reported questionnaire data and measurements (e.g. plasma cotinine) from the baseline were utilised. The outcome variable was smoking cessation at the follow-up, and the main explanatory variable was education. Logistic regression was the main method for statistical analyses. All of the analyses accounted for the sampling design. **Results:** At the follow-up, 28% of the baseline daily smokers had quit smoking. An adjusted regression model showed that highly educated respondents had a higher likelihood of quitting smoking compared with those with basic education. Controlling for demographic and health-related variables had a modest effect on this association. Higher scores for plasma cotinine, symptoms of depression and heavy alcohol use were associated with a lower likelihood of quitting smoking. The association between education and smoking cessation was weaker for women than it was for men. **Conclusions: High education is associated with smoking cessation among the general adult population, especially among men. A higher plasma cotinine level is strongly associated with continued smoking among both sexes. Background variables only modestly affected the association between education and smoking cessation.**

## Introduction

Smoking has a major influence on inequalities in health, as it is more prevalent among the less educated compared with the highly educated. Socio-economic differences in smoking have increased in Finland during the 2000s [1]. Thus, smoking cessation is pivotal in promoting public health and tackling inequalities in health between different population groups. Yet, the association between smoking cessation and education is understudied, especially among the general adult population, in a longitudinal study design. A more profound analysis of this is absent from earlier investigations on Northern European populations [2,3].

The reasons for socio-economic differences in smoking cessation might be the higher risk for relapse, lower motivation and less social support for quitting smoking, as well as the higher drop-out rate from smoking cessation treatment among lower socio-economic groups [4,5]. Additionally, lower socio-economic groups may face structural barriers, such as the poor approachability of or inability to pay for smoking cessation services [4].

There are studies in general populations on the predictors of smoking cessation [2], but none has focused on the role of education in particular. It is suggested that a higher level of education is associated with smoking cessation, but this association remains unclear [2,5,6]. A Finnish twin study found that a higher education predicted smoking cessation, even when smoking behaviour factors, such as the number of cigarettes smoked per day (CPD), and demographic variables were adjusted for [7]. A UK investigation examined the influence of demographic and cessation-related factors (such as the motivation to quit and social support for quitting) on the association between socio-economic position (SEP) and smoking cessation at different smoking cessation services [5]. SEP was found to influence smoking cessation, for example through social support and addiction [5]. For policy and practical implications, it is important to investigate which factors confound the association between education and smoking cessation among the general adult population.

Few predictors of smoking cessation are well established. Male gender and higher age may be associated with successful quitting, but the evidence is inconclusive [2,6,7]. People with symptoms of depression are less likely to quit smoking than those without such symptoms [3]. Also, alcohol use prevents smoking cessation [8]. A review including eight longitudinal studies on adult general populations found that only low cigarette dependence consistently predicted successful cessation [6]. A common proxy for cigarette dependence is the CPD [6]. A more reliable measure could be a biochemical marker, such as the plasma cotinine level [9].

The aim of this study was to examine the association between education and smoking cessation in a general adult population in an 11-year follow-up study. The study questions were:

(1) Is education associated with smoking cessation?
(2) To what extent do demographic and health-related factors affect the association between education and smoking cessation?
(3) Which demographic and health-related factors are associated with smoking cessation among the general adult population?

## Methods

### Data

The Health 2000 Survey is a longitudinal population-based study conducted during 2000–2001, based on two-stage stratified cluster sampling [10]. The sample size was 8028, and participants were aged ⩾30 years. Several methods were used, such as questionnaires, clinical examinations and determinations from blood samples. All the participants of the Health 2000 Survey who were alive, living in Finland and had not refused to take part in the study were invited to participate in the follow-up survey, Health 2011, between 2011 and 2012 (*N*=6319, aged ⩾41 years). Overall, the response rate for respondents aged ⩾41 years who participated in at least one data collection phase at the follow-up was 76% (*n*=4797) [11].

Our final analytic sample consisted of 1352 baseline daily smokers, of whom 945 reported a smoking status at the follow-up (response rate 70%). The final variable for smoking cessation included 884 respondents (occasional smokers at the follow-up omitted, *n*=61). The studies were approved by the Ethics Committee for Epidemiology and Public Health in the Hospital District of Helsinki and Uusimaa (Health 2000 Survey) and by the Coordinating Ethics Committee of the Hospital District of Helsinki and Uusimaa (Health 2011 Survey). All the participants gave their written informed consent.

### Variables

All of the information was self-reported, excluding the nurse-collected height and weight, composing body mass index (BMI) and plasma cotinine concentration [12]. Smoking status was assessed using three questions: ‘Have you ever smoked during your lifetime?’ (yes/no), ‘Have you smoked at least 100 times during your lifetime (cigarettes, cigars or pipes)?’ (yes/no) and ‘Do you currently smoke (cigarettes, cigars or pipes)?’ (daily/occasionally/no). Three mutually exclusive groups were identified: daily smokers, occasional smokers and non-smokers. The respondents were classified as daily smokers or occasional smokers if they had smoked during their lifetime and they currently smoked daily/occasionally. The respondents were classified as non-smokers if they had not smoked during their lifetime or if they had smoked during their lifetime but less than 100 times or if they did not smoke currently. The outcome variable was smoking cessation: those who reported daily smoking at the baseline and no smoking at the follow-up were classified as quitters of daily smoking.

In all analyses, baseline information on the demographic and health-related variables was used. The main explanatory variable in our analyses was educational level, taking into account both the number of educational years and the type of education/degree. Education was classified into three classes (basic, middle, high) based on the initial seven-classed variable containing information on both the basic education (from ‘less than elementary school’ to ‘matriculation examination’) and the highest level of education or the degree (from ‘no vocational training’ to ‘doctoral degree’) of the respondent. Covariates were included based on their earlier documented associations with smoking cessation. Demographic variables were age (continuous), gender (man/woman), employment status (employed/unemployed or laid off/retired/other or missing), marital status (living with a partner/living without a partner), under-aged children living in the household (none/at least one) and income per month weighted by the household size relative to the number of children (continuous). Health-related variables were CPD (continuous), cotinine **(μg/L)** (in quintiles, except continuous in Table I), alcohol consumption (g/week; no use: 0 g; moderate use – men <252 g, women <168 g; heavy use – men ⩾252 g, women ⩾168 g), self-perceived health (good/other), BMI (normal weight, including *n*=25 underweight: 25–29.99 kg/m^2^; overweight: 25–29.99 kg/m^2^; obese: ⩾30 kg/m^2^) and the Beck Depression Inventory [13] (none or minimal depression: 0–9 points; mild depression: 10–18 points; moderate or severe depression: 19–55 points).

**Table I.** Characteristics of baseline daily smokers by smoking status at the follow-up and their difference (*p*-value^a^), % or mean and *n.*^b^

	**Follow-up daily smokers (*N*=604)**			**Follow-up quitters (*N*=280)**			**Missing % (*n*)** ^c^
	**Men**	**Women**	**Total**	**Men**	**Women**	**Total**	
**Age (years), mean (*p*<0.0001)**	43.2	43.6	43.4	46.9	46.6	46.8	0 (0)
Total (*n*)	306	298	604	167	113	280	
**Gender distribution (*p*=0.029)**	54%	46%	100%	62%	39%	100%	0 (0)
Total (*n*)	306	298	604	167	113	280	
**Educational level (*p*=0.1078)**							0 (0)
Basic	40%	39%	40%	33%	41%	36%	
Middle	45%	37%	41%	44%	31%	39%	
High	16%	24%	19%	23%	29%	25%	
Total (*n*)	306	298	604	167	113	280	
**Employment status (*p*=0.0006)** ^d^							0 (0)
Employed	71%	73%	72%	73%	64%	70%	
Unemployed	16%	13%	15%	6%	13%	9%	
Retired	9%	8%	9%	18%	16%	18%	
Other/missing	4%	5%	4%	2%	7%	4%	
Total (*n*)	306	298	604	167	113	280	
**Marital status (*p*=0.0155)**							0 (0)
Living with a partner	63%	64%	64%	76%	66%	72%	
Living without a partner	37%	36%	36%	24%	34%	28%	
Total (*n*)	306	298	604	167	113	280	
**Under-aged children in the household (*p*=0.0228)**							0 (0)
None	56%	54%	55%	62%	67%	64%	
At least one	44%	46%	45%	38%	33%	36%	
Total (*n*)	306	298	604	167	113	280	
**Income per month, mean** ^e^ **(*p*=0.1258)**	93.06	93.28	93.17	106.68	90.34	100.42	5 (45)
Total (*n*)	289	286	566	164	109	273	
**Cigarettes per day, mean (*p*=0.0274)**	19.5	14.1	16.9	17.8	11.7	15.4	3 (27)
Total (*n*)	290	291	581	165	111	276	
**Plasma cotinine (µg/L), mean (*p*<0.0001)**	539.8	481.3	511.3	440.4	354.7	407.0	5 (40)
Total (*n*)	279	289	568	164	112	276	
**Alcohol consumption (*p*=0.0529)**							5 (41)
No use	14%	28%	21%	21%	31%	25%	
Moderate use	57%	60%	59%	61%	61%	61%	
Heavy use	28%	12%	20%	18%	8%	14%	
Total (*n*)	280	289	569	162	112	274	
**Self-perceived health (*p*=0.3264)**							0 (0)
Other	35%	29%	32%	41%	26%	35%	
Good	65%	71%	68%	59%	74%	65%	
Total (*n*)	306	298	604	167	113	280	
**BMI (*p*=0.0430)**							0 (0)
Normal weight	46%	50%	48%	37%	43%	39%	
Overweight	36%	31%	34%	47%	33%	42%	
Obese	17%	18%	18%	16%	23%	19%	
Total (*n*)	306	298	604	167	113	280	
**Depression symptoms** **(*p*=0.0022)**							7 (58)
None/minimal	73%	68%	71%	80%	77%	79%	
Mild	15%	19%	17%	17%	17%	17%	
Moderate/severe	12%	12%	12%	3%	6%	4%	
Total (*n*)	275	283	558	160	108	268	

a *p*-Value from the Wald test.

b Number of observations from the unweighted data.

c Number of missing observations and its percentage of the total number of follow-up smokers and follow-up quitters (*n*=884).

d The missing values are included in the class ‘other’ to maximise the number of observations in the analyses.

e Hundreds of euros.

BMI: body mass index.

### Statistical analyses

The characteristics of the baseline daily smokers by smoking status at the follow-up are presented in Table I, where the association between smoking status and background variables was tested using a regression model and with the Wald test. The chi-square test was used to examine the association between baseline education and discrete background variables, whereas linear regression was used to examine the association between baseline education (independent variable) and continuous covariates (dependent variables; Supplemental Table SI).

Bivariate and multiple binary logistic regression models, with 95% confidence intervals (CIs), were used to examine (a) the associations of the demographic and (b) health-related background variables and smoking cessation (Table II). Demographic variables were adjusted for in estimating the association between education and smoking cessation. Health-related variables were then included to investigate whether these variables explain the association between education and smoking cessation, when demographic variables are adjusted for. Model 1 was adjusted for age. Model 2 included additionally other demographic variables (gender, education, employment status, marital status, the number of under-aged children living in the household and income). Model 3 was further adjusted for health-related variables (CPD, plasma cotinine level, alcohol consumption, self-perceived health and BMI). For the final model (model 4), the measure for symptoms of depression was added. There was a statistically significant interaction between education and sex in the full model (*p*=0.0014). So, analyses stratified by gender were conducted (Tables III and IV). The magnitude with which the background variables explain the association between education and smoking cessation was assessed with a reformulated KHB method [14] (Supplemental Table SII).

**Table II.** Association between education and other baseline background variables with smoking cessation in the follow-up, odds ratios (OR) and their 95% confidence intervals (CI).

	**Model 1**		**Model 2**		**Model 3**		**Model 4**	
	**OR**	**95% CI**	**OR**	**95% CI**	**OR**	**95% CI**	**OR**	**95% CI**
***Demographic variables***								
**Educational level**								
Basic	1.00		1.00		1.00		1.00	
Middle	1.34	0.95–1.87	1.30	0.92–1.84	1.17	0.80–1.70	1.16	0.79–1.70
High	1.75**	1.22–2.50	1.81**	1.23–2.66	1.65*	1.08–2.53	1.62*	1.05–2.50
**Age**	N/A		1.03**	1.01–1.05	1.03**	1.01–1.05	1.03*	1.01–1.05
**Gender**								
Women	1.00		1.00		1.00		1.00	
Men	1.36*	1.03–1.80	1.50**	1.13–1.99	1.92***	1.39–2.64	1.83***	1.31–2.54
**Employment status**								
Employed	1.00		1.00		1.00		1.00	
Unemployed or laid off	0.57*	0.35–0.93	0.61	0.35–1.06	0.65	0.35–1.20	0.69	0.37–1.27
Retired	1.32	0.80–2.19	1.50	0.86–2.62	1.42	0.77–2.61	1.45	0.78–2.71
Other/missing	1.08	0.48–2.45	1.12	0.49–2.55	1.26	0.54–2.92	1.24	0.54–2.86
**Marital status**								
Living without a partner	1.00		1.00		1.00		1.00	
Living with a partner	1.39*	1.02–1.90	1.27	0.89–1.81	1.11	0.76–1.62	1.09	0.75–1.60
**Under-aged children in the household**								
None	1.00		1.00		1.00		1.00	
At least one	0.92	0.65–1.29	1.00	0.67–1.48	1.07	0.76–1.62	1.02	0.66–1.56
**Income per month**	1.00	1.00–1.00	1.00	1.00–1.00	1.00		1.00	1.00–1.00
***Health-related variables***								
**Cigarettes per day**	0.98*	0.96–1.00			0.98*	0.96–1.00	0.98	0.96–1.00
**Plasma cotinine** ^a^	0.47***	0.36–0.60			0.49***	0.38–0.63	0.48***	0.38–0.62
**Alcohol consumption**								
No use	1.00				1.00		1.00	
Moderate use	0.94	0.65–1.37			0.76	0.49–1.16	0.79	0.51–1.22
Heavy use	0.62*	0.40–0.97			0.44**	0.26–0.74	0.50*	0.29–0.85
**Self-perceived health**								
Other	1.00				1.00		1.00	
Good	1.02	0.77–1.35			0.94	0.68–1.29	0.84	0.60–1.18
**BMI**								
Normal weight	1.00				1.00		1.00	
Overweight	1.42*	1.03–1.95			1.37	0.97–1.94	1.39	0.98–1.97
Obese	1.13	0.75–1.70			1.19	0.75–1.86	1.18	0.75–1.87
**Depression symptoms**								
None/minimal	1.00						1.00	
Mild	0.81	0.53–1.22					0.74	0.47–1.15
Moderate/severe	0.29***	0.15–0.57					0.35**	0.17–0.73

Model 1: adjusted for age (estimates of the background variables from bivariate analysis including age).

Model 2: Model 1+adjusted for education, gender, employment status, number of under-aged children living in the household and income.

Model 3: Model 2+adjusted for cigarettes per day, plasma cotinine level, alcohol consumption, self-perceived health and BMI.

Model 4: Model 3+adjusted for symptoms of depression (all the background variables in the model).

Bold indicates statistical significance (*p*<0.05).

a OR per an increment of one quintile; the cut-off points were 2, 7, 14 and 220.

* *p*<0.05; **p<0.01; ***p<0.001.BMI: body mass index.

**Table III.** Association between education and other baseline background variables with smoking cessation in the follow-up, men, OR and their 95% confidence intervals (CI).

	**Model 1**		**Model 2**		**Model 3**		**Model 4**	
	**OR**	**95% CI**	**OR**	**95% CI**	**OR**	**95% CI**	**OR**	**95% CI**
***Demographic variables***								
**Educational level**								
Basic	1.00		1.00		1.00		1.00	
Middle	1.56	1.00–2.44	1.52	0.97–2.38	1.52	0.91–2.52	1.49	0.89–2.48
High	2.01**	1.22–3.32	1.99*	1.15–3.43	2.12*	1.12–3.99	2.08*	1.09–3.98
**Age**	N/A		1.03	1.00–1.06	1.03	0.99–1.06	1.03	1.00–1.07
**Employment status**								
Employed	1.00		1.00		1.00		1.00	
Unemployed or laid off	0.35**	0.17–0.73	0.35*	0.15–0.82	0.41*	0.17–0.97	0.44	0.19–1.01
Retired	1.23	0.63–2.42	1.75	0.77–4.00	1.69	0.68–4.20	1.75	0.69–4.40
Other/missing	0.57	0.15–2.24	0.62	0.16–2.46	0.96	0.21–4.38	0.82	0.14–4.76
**Marital status**								
Living without a partner	1.00		1.00		1.00		1.00	
Living with a partner	1.62*	1.02–2.55	1.15	0.63–2.09	0.97	0.49–1.93	0.86	0.44–1.67
**Under-aged children in the household**								
None	1.00		1.00		1.00		1.00	
At least one	1.03	0.67–1.58	1.19	0.67–2.13	1.50	0.77–2.94	1.53	0.79–2.97
**Income per month**	1.00	1.00–1.01	1.00	1.00–1.01	1.00	1.00–1.01	1.00	1.00–1.01
***Health-related variables***								
**Cigarettes per day**	0.98*	0.96–1.00			0.99	0.96–1.01	0.99	0.96–1.01
**Plasma cotinine**	0.54***	0.41–0.71			0.55***	0.41–0.74	0.53***	0.40–0.72
**Alcohol consumption**								
No use	1.00				1.00		1.00	
Moderate use	0.82	0.46–1.45			0.50*	0.26–0.95	0.52	0.27–1.00
Heavy use	0.47*	0.25–0.87			0.27***	0.13–0.54	0.30**	0.14–0.61
**Self-perceived health**								
Other	1.00				1.00		1.00	
Good	0.89	0.61–1.30			0.70	0.44–1.12	0.65	0.40–1.05
**BMI**								
Normal weight	1.00				1.00		1.00	
Overweight	1.54*	1.02–2.35			1.55	0.95–2.52	1.64	1.00–2.68
Obese	1.06	0.60–1.87			1.05	0.54–2.04	1.10	0.57–2.12
**Depression symptoms**								
None/minimal	1.00						1.00	
Mild	0.93	0.52–1.64					0.79	0.42–1.49
Moderate/severe	0.23**	0.09–0.59					0.28*	0.10–0.81

Model 1: adjusted for age (estimates of the background variables from bivariate analysis including age).

Model 2: Model 1+adjusted for education, employment status, number of under-aged children living in the household and income.

Model 3: Model 2+adjusted for cigarettes per day, plasma cotinine level, alcohol consumption, self-perceived health and BMI.

Model 4: Model 3+adjusted for symptoms of depression (all the background variables in the model).

Bold indicates statistical significance (*p*<0.05).

a OR per an increment of one quintile; the cut-off points were 2, 7, 14 and 220.

* *p*<0.05; **p<0.01; ***p<0.001.

BMI: body mass index.

Statistical software packages Stata/SE v16.0 (StataCorp, College Station, TX) and IBM SPSS Statistics for Windows v25 (IBM Corp., Armonk, NY) were used in data management and analyses. The sampling design and inverse probability weights to handle non-response and oversampling were accounted for in all of the analyses using the survey procedure. Analyses included all the available observations (no list-wise deletion) except for the KHB analysis, which in every step included only the same observations (those with no missing values in all the variables included in the analyses).

Drop-out analyses (unweighted) revealed that of the follow-up non-participants, 47% had basic education compared with 36% of the follow-up respondents (not shown). Higher education was associated with a higher probability of participation, while older age and male gender were associated with a lower probability of participation at the follow-up (Supplemental Table SIII). The missing data analyses in the Health 2000 Survey and Health 2011 Survey have been examined in detail elsewhere [15].

## Results

### Descriptive analyses

At baseline, 21% of the population smoked daily (26% of men and 17% of women). During the follow-up, 28% of the baseline daily smokers had quit smoking (31% of men, 25% of women). The proportion of highly educated respondents tended to be greater among follow-up quitters than among follow-up smokers, although this was statistically non-significant (Table I). The quitters were more likely to be older and men, and they also had lower scores for cotinine and CPD than smokers did. Symptoms of depression were less prevalent among follow-up quitters. Women were more highly educated than men were, and the cotinine score and CPD had an inverse association with education (Supplemental Table SI).

### Multiple adjusted analyses

In the age-adjusted model, those with higher education had a higher probability of smoking cessation than those with basic education did (Table II, model 1). Adjusting for demographic variables and health-related variables had a negligible effect on the association between education and smoking cessation (models 2 and 3). Further adjustment for depression symptoms attenuated this association only slightly (model 4). Thus, the association between education and smoking cessation remained significant through the adjustments.

In the final model (model 4), male gender and older age were associated with smoking cessation, whereas higher serum concentrations of cotinine, heavy use of alcohol and moderate or severe symptoms of depression were associated with a lower likelihood of smoking cessation. These associations were quite robust across the models.

All the background variables decreased the effect of high and middle education on smoking cessation by 20% and 42%, respectively (Supplemental Table SII). Health-related variables decreased the effect of high education on smoking cessation by 19% and of middle education by 17%.

Men with higher education had a greater likelihood of smoking cessation, but for women, the association was weaker (Tables III and IV). Among men, higher plasma cotinine level, heavy use of alcohol and moderate or severe symptoms of depression predicted a lower probability of smoking cessation (Table III, model 4). Among women, plasma cotinine only showed a statistically significant association with smoking cessation (Table IV, model 4).

**Table IV.** Association between education and other baseline background variables with smoking cessation in the follow-up, women, OR and their 95% confidence intervals (CI).

	**Model 1**		**Model 2**		**Model 3**		**Model 4**	
	**OR**	**95% CI**	**OR**	**95% CI**	**OR**	**95% CI**	**OR**	**95% CI**
***Demographic variables***								
**Educational level**								
Basic	1.00		1.00		1.00		1.00	
Middle	0.98	0.60–1.62	1.02	0.59–1.75	0.91	0.51–1.62	0.94	0.52–1.71
High	1.52	0.84–2.74	1.75	0.93–3.27	1.40	0.74–2.65	1.41	0.74–2.69
**Age**	N/A		1.03	0.99–1.06	1.03	0.99–1.06	1.02	0.99–1.06
**Employment status**								
Employed	1.00		1.00		1.00		1.00	
Unemployed or laid off	1.05	0.55–1.97	1.15	0.57–2.31	1.18	0.52–2.68	1.21	0.52–2.82
Retired	1.40	0.65–3.02	1.36	0.57–3.20	1.30	0.51–3.32	1.23	0.45–3.31
Other / missing	1.90	0.66–5.51	1.74	0.60–5.02	1.72	0.58–5.13	1.67	0.57–4.87
**Marital status**								
Living without a partner	1.00		1.00		1.00		1.00	
Living with a partner	1.12	0.71–1.78	1.32	0.80–2.18	1.06	0.62–1.80	1.16	0.67–2.00
**Under-aged children in the household**								
None	1.00		1.00		1.00		1.00	
At least one	0.75	0.43–1.28	0.78	0.45–1.34	0.72	0.40–1.29	0.66	0.36–1.18
**Income per month**	1.00	1.00–1.00	1.00	0.99–1.00	1.00	0.99–1.00	1.00	0.99–1.00
***Health-related variables***								
**Cigarettes per day**	0.95**	0.92–0.98			0.97*	0.94–1.00	0.97	0.94–1.00
**Plasma cotinine**	0.34***	0.22–0.54			0.42**	0.26–0.69	0.42**	0.26–0.69
**Alcohol consumption**								
No use	1.00				1.00		1.00	
Moderate use	0.95	0.59–1.54			0.96	0.56–1.66	1.04	0.59–1.83
Heavy use	0.68	0.29–1.61			0.79	0.31–1.98	0.95	0.37–2.45
**Self-perceived health**								
Other	1.00				1.00		1.00	
Good	1.38	0.82–2.30			1.34	0.74–2.41	1.23	0.66–2.28
**BMI**								
Normal weight	1.00				1.00		1.00	
Overweight	1.17	0.70–1.95			1.11	0.64–1.92	1.09	0.63–1.90
Obese	1.25	0.69–2.23			1.34	0.72–2.49	1.30	0.68–2.47
**Depression symptoms**								
None/minimal	1.00						1.00	
Mild	0.72	0.42–1.25					0.72	0.40–1.29
Moderate/severe	0.40	0.16–1.02					0.47	0.17–1.29

Model 1: adjusted for age (estimates of the background variables from bivariate analysis including age).

Model 2: Model 1+adjusted for education, employment status, number of under-aged children living in the household and income.

Model 3: Model 2+adjusted for cigarettes per day, plasma cotinine level, alcohol consumption, self-perceived health and BMI.

Model 4: Model 3+adjusted for symptoms of depression (all the background variables in the model).

Bold indicates statistical significance (*p*<0.05).

a OR per an increment of one quintile; the cut-off points were 2, 7, 14 and 220.

* *p*<0.05; **p<0.01; ***p<0.001.

BMI: body mass index.

## Discussion

Our results regarding a nationally representative general adult population follow-up study show that highly educated men were more likely to quit smoking than less educated men. This association remained after taking several demographic and health-related background factors into account. In particular, higher scores for plasma cotinine level (indicating high nicotine dependence), symptoms of depression and heavy use of alcohol were associated with a lower probability of smoking cessation. In women, the association between education and smoking cessation was somewhat parallel to that of men but weaker, and it failed to reach statistical significance.

Earlier studies show that education may be associated with smoking cessation [6,7]. Consistent with our results, a study from northern Europe (excluding Finland) found that the higher educated were more likely to quit smoking [2]. The explanatory effect of sociodemographic and health-related variables on the association between education and smoking cessation has not been examined in previous studies. In our investigation, a large part of this association remained unexplained by the included variables, implying a strong association between education and smoking cessation, especially among men.

Male gender and to some extent older age were associated with the higher likelihood of smoking cessation, which supports some earlier findings yet contradicts others [2,6]. Inconsistent results might be due to, for example, differences in the study populations or the current state of the tobacco epidemic [16]. Our results imply that women have more difficulties in quitting smoking compared with men. Social/cultural and temporal factors may play a role in the mixed evidence from observational studies examining gender differences in smoking cessation [17]. It might be that in Finland, women are less likely either to seek or to receive treatment, or there might be motivational differences considering smoking cessation [18]. However, this has not been studied among Finnish adults. Only a few factors were associated with smoking cessation among women. This supports an earlier finding that educational differences are less pronounced among women regarding smoking [1]. In addition, the number of respondents were limited in the stratified analyses (unweighted *n*=473 for men; unweighted *n*=411 for women), which may explain some of the statistically non-significant findings.

Prior studies have shown that nicotine dependence is associated with smoking cessation [3,6,7]. General adult population studies have predominantly utilised a subjective measure of dependence [6]. In our investigation, the plasma cotinine level (an objective measure) showed a stronger and more stable association with smoking cessation than CPD (a subjective measure). Additional analyses showed that both CPD and the cotinine level were statistically significantly associated with smoking cessation in the fully adjusted model when the other one was excluded. Still, the association between the cotinine level and smoking cessation was stronger (for men, only the cotinine level reached statistical significance; not shown). The objective measure takes into account better the individual-level factors affecting nicotine dependence, such as differences in inhalation and metabolism [19]. Misreporting might also occur with self-reports. Thus, the objective measures of dependence could be seen as more reliable than the subjective measures of dependence.

Respondents with moderate or severe symptoms of depression had a smaller likelihood of smoking cessation than those with fewer or no symptoms. This finding is supported by another general adult population study utilising Finnish twin data [3]. Smoking cessation is not associated with an increase in depression among those with a past history of depression, and subjective well-being increases after quitting smoking [20,21]. In the current study, the association between symptoms of depression and smoking cessation was quite robust. Together, these results highlight that depression plays a major role in smoking cessation, and smoking cessation could be viewed as a remedial as well as preventive action in health-care systems relating to depression.

In addition to individual characteristics, policy actions may encourage or hinder smoking cessation. Smokers are more likely to quit in countries where tobacco control policy is stricter [22]. Finland has a strict tobacco control policy, which is largely supported by the population [23]. Yet, supporting smokers in their efforts to quit has been one of the weakest points of the Finnish tobacco control policy [24]. Tax increases reduce inequalities in smoking by affecting the lower SEP smokers more (equity-positive impact) [25]. Several tobacco tax increases have been implemented in Finland since 2009. The observed differences in smoking cessation between educational groups may have been less pronounced if there had been more tax increases during the study period. A systematic review has identified zero equity-positive, individual-level smoking cessation interventions [26]. However, there are implications that technology-based interventions, such as websites for cessation, may reduce smoking more among the lower SEP groups [27].

Finland aims to be tobacco and nicotine free by 2030 [28]. Our results, alongside findings from the earlier investigations showing widening educational differences in tobacco use [1,29], suggest that supporting the less educated is pivotal if this objective is to be reached. Enhanced smoking cessation support should also be targeted at highly addicted smokers, as well as at those with at least moderate symptoms of depression. Quitting treatment may be more likely among the lower SEP groups [5], which should be considered when developing cessation services.

### Limitations and strengths

This study has some limitations. The design prevents any causal associations between education and smoking cessation from being studied. Higher education was associated with the higher probability of response at the follow-up compared with basic education, indicating possible bias due to attrition. However, education was included as one component of the inverse probability weights which reduces the effect of lower participation among the less educated. The weights also correct for the different participation rates in different age groups and genders [15]. Overall, the effect of the drop-out on the results is modest. The presented results are conservative estimates for the educational differences: the differences could have been more pronounced had the less educated responded more actively. Smoking status was self-reported, and underestimation of smoking might have occurred. Still, a self-reported smoking status is fairly accurate and does not vary by SEP [30]. Future studies should also include systemic variables to account for the possible effects of societal factors on the association between education and smoking cessation.

The strengths of our study are numerous. A rarely utilised longitudinal design from a population-based sample with inverse probability weights and a good response rate was used. A biochemical measure was included as a proxy for nicotine dependence at the baseline. This proved to be a superior predictor of smoking cessation than CPD– a more commonly used measure in general population studies. The possibility that some underlying mechanisms affect the association between education and smoking cessation cannot be ruled out. However, several demographic and health-related background variables could be taken into account when examining this association. The results from the pooled analyses (men and women together) are generalisable to the Finnish adult population.

## Conclusions

This investigation showed that a higher educational level is longitudinally associated with smoking cessation in a general adult population sample. Some health-related factors, especially higher plasma cotinine level, are associated with a lower likelihood of smoking cessation. To tackle inequalities in health, enhanced support for quitting smoking should be provided, especially for the less educated. If the objective of a tobacco-free Finland by 2030 is to be reached, differences between educational groups in smoking cessation need to be eradicated.

## Supplemental Material

sj-pdf-1-sjp-10.1177_1403494821993721 – Supplemental material for Association between educational level and smoking cessation in an 11-year follow-up study of a national health survey Click here for additional data file. Supplemental material, sj-pdf-1-sjp-10.1177_1403494821993721 for Association between educational level and smoking cessation in an 11-year follow-up study of a national health survey by Otto Ruokolainen, Tommi Härkänen, Jouni Lahti, Ari Haukkala, Markku Heliövaara and Ossi Rahkonen in Scandinavian Journal of Public Health

sj-pdf-2-sjp-10.1177_1403494821993721 – Supplemental material for Association between educational level and smoking cessation in an 11-year follow-up study of a national health survey Click here for additional data file. Supplemental material, sj-pdf-2-sjp-10.1177_1403494821993721 for Association between educational level and smoking cessation in an 11-year follow-up study of a national health survey by Otto Ruokolainen, Tommi Härkänen, Jouni Lahti, Ari Haukkala, Markku Heliövaara and Ossi Rahkonen in Scandinavian Journal of Public Health

sj-pdf-3-sjp-10.1177_1403494821993721 – Supplemental material for Association between educational level and smoking cessation in an 11-year follow-up study of a national health survey Click here for additional data file. Supplemental material, sj-pdf-3-sjp-10.1177_1403494821993721 for Association between educational level and smoking cessation in an 11-year follow-up study of a national health survey by Otto Ruokolainen, Tommi Härkänen, Jouni Lahti, Ari Haukkala, Markku Heliövaara and Ossi Rahkonen in Scandinavian Journal of Public Health
