# Supplementary material for: Association between educational level and smoking cessation in an 11-year follow-up study of a national health survey
Source: Scand J Public Health. 2021 Mar 1;49(8):951–60. doi: 10.1177/1403494821993721 (PMC8573358; doi:10.1177/1403494821993721)
Supplement: sj-pdf-1-sjp-10.1177_1403494821993721 – Supplemental material for Association between educational level and smoking cessation in an 11-year follow-up study of a national health survey [file sj-pdf-1-sjp-10.1177_1403494821993721.pdf]

Supplementary Table I. Association between education and discrete background variables, %, (Panel A) and continuous covariates (Panel B) among baseline daily smokers.

PANEL A: DISCRETE BACKGROUND VARIABLES

	Low	Middle	High	p-value[a]
**Gender**				0.0096
Men	37 %	44 %	19 %	
Women	39 %	35 %	26 %	
**Employment status**				<0.001
Employed	58 %	77 %	82 %	
Unemployed or laid off	18 %	12 %	8 %	
Retired	21 %	7 %	5 %	
Other / missing	4 %	4 %	5 %	
**Marital status**				0.8055
Living with a partner	67 %	66 %	69 %	
Living without a partner	33 %	34 %	31 %	
**Under-aged children in the household**				<0.001
None	66 %	49 %	53 %	
At least one	34 %	51 %	47 %	
**Alcohol consumption**				0.0008
No use	30 %	20 %	14 %	
Moderate use	55 %	61 %	64 %	
Heavy use	15 %	19 %	22 %	
**Self-perceived health**				<0.001
Other	45 %	25 %	27 %	
Good	55 %	75 %	73 %	
**BMI**				0.8570
Normal weight	45 %	47 %	46 %	
Overweighted	36 %	37 %	36 %	
Obese	19 %	16 %	18 %	
**Symptoms of depression**				0.0544
None/minimal	68 %	77 %	76 %	
Mild	20 %	15 %	18 %	
Moderate/severe	13 %	8 %	7 %	

PANEL B: CONTINUOUS COVARIATES[b]

	Coefficicient	95 % CI	p-value
**Age**	-2.571	-3.42, (-1.72)	<0.001
**Income per month**	19.65	14.48-24.83	<0.001
**Cigarettes per day**	-1.554	-2.27, (-0.83)	<0.001
**Plasma cotinine**	-0.086	-0.14, (-0.03)	0.002

[a]: P-value from Chi-squared test

[b]: Coefficients from linear regression, covariate as the dependent variable and education as the independent variable
