# Supplementary material for: Association between educational level and smoking cessation in an 11-year follow-up study of a national health survey
Source: Scand J Public Health. 2021 Mar 1;49(8):951–60. doi: 10.1177/1403494821993721 (PMC8573358; doi:10.1177/1403494821993721)
Supplement: sj-pdf-2-sjp-10.1177_1403494821993721 – Supplemental material for Association between educational level and smoking cessation in an 11-year follow-up study of a national health survey [file sj-pdf-2-sjp-10.1177_1403494821993721.pdf]

Supplemental Table SII. Logistic models using linear predictor method (Breen et al. 2018), regressing smoking cessation on education and background variables/covariates. Only estimates for education are displayed (Panel A). Mediated proportions of the association between education and smoking cessation by education (Panel B).

Panel A. Logistic models, only estimates for education are displayed.

**Educational level (ref. Basic)**	Model 1	Model 2	Model 3	Model 4	Model 5	Model 6	Model 7	Model 8	Model 9	Model 10	Model 11	Model 12
Middle	0.254	0.204	0.187	0.178	0.171	0.178	0.146	0.120	0.148	0.149	0.139	0.147
High	0.600	0.622	0.585	0.579	0.578	0.595	0.515	0.417	0.496	0.497	0.482	0.483

All models adjusted for age; Model 1 including education; Model 2: Model 1+ gender; Model 3: Model 2 + employment status; Model 4: Model 3 + marital status; Model 5: Model 4 + under-aged children in the household; Model 6: Model 5 + income; Model 7: Model 6 + cigarettes per day; Model 8: Model 7 + plasma cotinine level; Model 9: Model 8 + alcohol consumption; Model 10: Model 9 + self-reported health; Model 11: Model 10 + BMI; Model 12: Model 11 + symptoms of depression.

Panel B. Mediated proportions of the association between education and smoking cessation.

**Educational level (ref. Basic)**	Total (M12 vs M1)	Demographic (M1 vs M6)	Health (M6 vs M12)
Middle	42 %	30 %	17 %
High	20 %	1 %	19 %

Abbreviations, e.g. M_n, refer to model number in Panel A.

Total: total model including demographic and health-related variables; Demographic = Model including only demographic variables; Health = Model including only health-related variables.

KHB analyses: the confounding effect of the background variables on the association between education and smoking cessation

The analyses are based on Breen et al. (2018). Comparing the estimates of educational groups between models, the confounding effect of covariates/background variables on the association between the main explanatory variable (education) and outcome (smoking cessation) can be analysed. In this, the term ‘confound’ is being used instead of ‘mediate’, to imply less causal associations among the variables (see MacKinnon et al. 2000).

The confounding effect of demographic variables and health-related variables on the association between education and smoking cessation can be calculated as follows (for high education): 1-(estimate at Model 12/estimate at Model 1) = 1-(0.483/0.600) = 0.195. Thus, adding demographic and health-related variables reduces the effect of high education by 20% on the association between education and smoking cessation.

The confounding effect of demographic variables can be calculated as follows (for high education): 1-(estimate at Model 6/estimate at Model 1) = 1-(0.595/0.600) = 0.008. Thus, adding demographic variables reduces the effect of high education by 1% on the association between education and smoking cessation.

The confounding effect of health-related variables can be calculated as follows (for high education): 1-(estimate at Model 12/estimate at Model 6) = 1-(0.483/0.595) = 0.188. Thus, adding health-related variables reduces the effect of high education by 19% on the association between education and smoking cessation.

References:

Breen R, Karlson KB, Holm A. A note on a reformulation of the KHB method. Published online August 1st. *Sociol Method Res* 2018.

MacKinnon DP, Krull JL, Lockwood CM. Equivalence of the mediation, confounding and suppression effect. *Prev Sci* 2000;1:173.
