# Supplementary material for: Association between educational level and smoking cessation in an 11-year follow-up study of a national health survey
Source: Scand J Public Health. 2021 Mar 1;49(8):951–60. doi: 10.1177/1403494821993721 (PMC8573358; doi:10.1177/1403494821993721)
Supplement: sj-pdf-3-sjp-10.1177_1403494821993721 – Supplemental material for Association between educational level and smoking cessation in an 11-year follow-up study of a national health survey [file sj-pdf-3-sjp-10.1177_1403494821993721.pdf]

Supplemental Table SIII. Baseline characteristics of *non-respondents* at the follow-up (Panel A), and univariate association (Panel B) and multivariable association (Panel C) between *participation* at the follow-up and baseline background variables, odds ratios (OR) and their 95 % confidence intervals (95% CI).

	PANEL A	PANEL B[a]		PANEL C[b]	
	% (n)	OR	95% CI	OR	95% CI
DEMOGRAPHIC VARIABLES					
**Educational level**					
Basic	47% (189)	1.00		1.00	
Middle	40% (161)	**1.31***	1.01, 1.69	1.12	0.85, 1.47
High	14% (54)	**2.32*****	1.65, 3.29	**1.58***	1.11, 2.26
**Age (mean)**	45.9 (407)	0.99	0.98, 1.00	**0.98****	0.96, 0.99
**Gender**					
Women	37% (150)	1.00		1.00	
Men	63% (257)	**0.67****	0.53, 0.85	**0.56*****	0.43, 0.73
**Employment status**					
Employed	58% (237)	1.00		1.00	
Unemployed or laid off	18% (73)	**0.56*****	0.40, 0.77	0.77	0.52, 1.14
Retired	20% (81)	**0.43*****	0.31, 0.60	**0.37*****	0.24, 0.57
Other / missing	4% (16)	0.80	0.44, 1.46	0.66	0.35, 1.22
**Marital status**					
Living without a partner	37% (152)	1.00		1.00	
Living with a partner	63% (255)	1.26	0.99, 1.60	**1.56****	1.19, 2.04
**Under-aged children in the household**					
None	57% (230)	1.00		1.00	
At least one	43% (177)	0.91	0.72, 1.14	**0.71***	0.52, 0.95
**Income per month (mean)**	84.60 (351)	**1.00*****	1.00, 1.01	1.00	1.00, 1.00
HEALTH-RELATED VARIABLES					
**Cigarettes per day (mean)**	17.5 (357)	**0.98****	0.97, 0.99	0.99	0.98, 1.01
**Plasma cotinine[c] (mean)**	495.3 (315)	0.92	0.75, 1.15	0.93	0.80, 1.08
**Alcohol consumption**					
No use	26% (86)	1.00		1.00	
Moderate use	52% (171)	**1.42***	1.04, 1.92	0.97	0.71, 1.31
Heavy use	22% (73)	0.95	0.65, 1.38	0.81	0.55, 1.19
**Self-perceived health**					
Other	42% (170)	1.00		1.00	
Good	58% (237)	**1.53*****	1.21, 1.95	1.27	0.98, 1.64
**BMI**					
Normal weight	41% (168)	1.00		1.00	
Overweight	37% (152)	0.90	0.69, 1.17	0.83	0.63, 1.08
Obese	21% (87)	0.77	0.56, 1.05	0.77	0.56, 1.06
**Symptoms of depression**					
None/minimal	71% (214)	1.00		1.00	
Mild	21% (61)	0.79	0.57, 1.11	0.81	0.60, 1.09
Moderate/severe	9% (26)	0.93	0.58, 1.49	1.17	0.75, 1.82

[a]: Univariate association between response at the follow-up and baseline background variable

[b]: Multivariable association between response at the follow-up and baseline background variables; all the variables included simultaneously in the model

[c]: Continuous in Panel A, in quintiles in Panel B and Panel C. OR per an increment of one quintile; the cut-off points were 2, 7, 14, and 220

Bold typeface indicates statistical significance ($p<0.05$)

* $p<0.05$, **$p<0.01$, ***$p<0.001$
